# Impact of infection-related admission in patients with heart failure: a 10 years national cohort study

**DOI:** 10.1038/s41598-023-34028-8

**Published:** 2023-04-28

**Authors:** Chao-Yu Chen, Cheng-Han Lee, Hui-Wen Lin, Sheng-Hsiang Lin, Yi-Heng Li

**Affiliations:** 1 Division of Cardiology, Department of Internal Medicine, National Cheng Kung University Hospital, College of Medicine, National Cheng Kung University, 138 Sheng Li Road, Tainan, Taiwan; 2 Department of Pharmacy, Institute of Clinical Pharmacy and Pharmaceutical Sciences, College of Medicine, National Cheng Kung University, Tainan, Taiwan; 3 Biostatistics Consulting Center, National Cheng Kung University Hospital, College of Medicine, National Cheng Kung University, Tainan, Taiwan; 4 Institute of Clinical Medicine, College of Medicine, National Cheng Kung University, Tainan, Taiwan; 5 Department of Public Health, College of Medicine, National Cheng Kung University, Tainan, Taiwan

**Keywords:** Cardiology, Disease prevention

## Abstract

Infection is a common cause of hospitalization in patients with heart failure (HF). The impact of infection on long term cardiovascular outcome in HF is not well studied. The aim of this study was to compare the long term risk of major adverse cardiovascular events (MACE) in HF patients with or without prior hospitalization for infection. From 2009 to 2015, 310,485 patients with their first HF admissions were enrolled from the Taiwan National Health Insurance Research Database. Among the patients, those with readmission due to infection within one year after HF discharge were defined as infection group and those without any infection admission were controls. The propensity score matching method was used to balance covariates between the two groups. Patients were followed until the occurrence of any component of the MACE or the end date of the study, December 31, 2019. In a mean follow-up time of 4.29 ± 2.92 years, 86.19% of patients in the infection group and 63.63% of patients in the control group had MACE. Multivariate Cox proportional hazards analysis showed the infection group had a higher risk of MACE (HR 1.760, 95% CI 1.714–1.807), including all-cause mortality (HR 1.587, 95% CI 1.540–1.636), myocardial infarction (HR 1.332, 95% CI 1.224–1.450), stroke (HR 1.769, 95% CI 1.664–1.882) and hospitalization for HF (HR 1.993, 95% CI 1.922–2.066). In conclusion, many HF patients discharged from the hospital experienced acute infection that required readmission. The patients had worse cardiovascular outcome after readmission for infectious disease compared to those without any infection.

## Introduction

Heart failure (HF) is a growing pandemic with high risks of hospitalization and mortality worldwide^1^. In a US retrospective cohort, the mean hospitalization rate was 1.3 per person-year^2^. The high annual hospitalization and mortality rate cause a great health and economic burden^3^. The in-hospital mortality of HF was around 3.9 to 6.4% across different region^4,5^. A national cohort study in Asia showed the mortality rate at 90 days, one year and three years after index HF admission were 8.9%, 22.5% and 42.8%, respectively^6^. Even if the patients could be discharged successfully, some of them still have unresolved symptoms and carry elevated risk of mortality. Although the guideline-directed medical therapies (GDMT) are more widely adopted nowadays, the mortality rate of HF is still worse than those with some malignancies without HF^7,8^.

Infection is one of the major precipitating factors of HF decompensation requiring hospitalization. In OPTIMIZE-HF (Organized Program to Initiate Lifesaving Treatment in Hospitalized Patients With Heart Failure) registry including admitted HF patients irrespective of their ventricular function, 15.3% of hospitalization for HF (HHF) were related to pneumonia or respiratory tract infection^9^. Infection is not only a driver of HHF, it also contributes to the worsening of cardiovascular outcome. In a prospective observational cohort study of 711 patients with heart failure with reduced ejection fraction (HFrEF), about one-fourth of their first hospitalizations were due to infection. After a longest 48-month follow-up, the mortality risk was significantly higher in patients with infection than those without infection and the mortality risk after infection-related discharge was similar to those after decompensated HF hospitalization^10^. The impact of infection on clinical outcome was not only observed in HFrEF, but also in patients with heart failure with preserved ejection fraction (HFpEF). In the post-hoc analyses of the patients with HFrEF in the PARADIGM-HF (Prospective Comparison of Angiotensin Receptor-Neprilysin Inhibitor With Angiotensin Converting Enzyme Inhibitor to Determine Impact on Global Mortality and Morbidity in Heart Failure) and patients with HFpEF in the PARAGON-HF (Prospective Comparison of ARNI with ARB Global Outcomes in Heart Failure with Preserved Ejection Fraction) trials, there was a twofold higher risk of HHF and a fourfold higher of mortality after an incident pneumonia^11^. The hazard ratio (HR) for the risk of all-cause mortality after pneumonia was similar between HFrEF in PARADIGM-HF (HR 4.34, 95% confidence interval [CI] 3.73 to 5.05) and HFpEF in PARAGON-HF (HR 3.76, 95% CI 3.09 to 4.58). In both HFrEF and HFpEF groups, the increased risk of mortality was extremely high in the first month after pneumonia and the risk sustained after 3 months after the episode of pneumonia^11^. These studies have demonstrated that infection not only acts as an acute trigger for the deterioration of HF but also carries negative impact on cardiovascular (CV) outcome. Therefore, vaccination and other measures for infection prevention have been widely recommended in major HF guidelines^12,13^. Clinically, it is common to assume that the effects of infection usually occur during the acute stage and dissipates in a period of time after the invading organisms are got rid of by the immune system and treatment. However, some infections could induce persistently elevated levels of inflammation after the acute episode and cause long-term problems. The long-term CV outcomes in HF after an infection episode have not been well studied. Our study aimed to track the long-term CV outcomes up to 10 years and demonstrated the negative legacy effects of infection in HF patients.

## Methods

### Data source

This is a nationwide, retrospective cohort study and all data were retrieved from the Taiwan National Health Insurance Research Database (NHIRD). The Taiwan National Health Insurance is a universal mandatory coverage, single-payer system established in 1995 and this system covers 99.5% of the 23 million residents in Taiwan. NHIRD is derived from National Health Insurance program’s claim data and allows complete tracking all records of demographic data, medical diagnosis, outpatient visits, hospitalizations, and medications and procedures used in inpatient and outpatient services. The International Classification of Diseases, Ninth Revision, Clinical Modification (ICD-9-CM) codes or, after 2016 the Tenth Revision (ICD-10-CM) were used to identify diagnosis (Supplementary Table S1). All personal information remained encrypted to keep the study participants’ privacy. There were validation studies on NHIRD that confirm this database is a valid resource for population research in cardiovascular diseases diagnosis^14^. The study was conducted according to the principles expressed in the Declaration of Helsinki and was approved by the Institutional Review Board of the National Cheng Kung University Hospital, Taiwan (IRB No: A-EX-111–011). The National Cheng Kung University Hospital Institutional Review Board granted a waiver of informed consent due to de-identified data and retrospective nature.

### Study design

From January 1, 2009 to December 31, 2015, all adult patients (≥ 18 years) who were hospitalized and survived to discharge with a primary diagnosis of HF were included in the study. The exclusion criteria were those who were younger than 18 years of age, had a previous outpatient or inpatient diagnosis of HF within one year, or had incomplete medical records. After enrollment, we followed up these HF patients to see if there was any readmission due to infectious disease within one year after HF discharge. We then divided the cohort into two groups: the infection group with an infection-related readmission within one year after HF discharge, and the control group with no history of infection-related readmission. For infection group, patients with a major discharge diagnosis of infectious diseases, including pneumonia, urinary tract infection (UTI), abscess, cellulitis, retroperitoneal infection, necrotizing fasciitis, infective endocarditis and sepsis, were included. The patients who died during hospitalization or within 30 days of discharge after infection-related admission were excluded. To access the impact of different sites of infections on CV outcomes, those with mixed site infections during admission were also excluded. The date of discharge after infection-related admission was used as the index date for further follow-up. For control group, we matched 1:1 with the study group for the index year, which is defined as the year of the patient's first hospitalization and diagnosis of heart failure. We then determined the middle date of the index year and used it as the dummy infection discharge date for the control group.

### Follow-up and endpoints

The primary outcome was a composite endpoint of all-cause mortality, myocardial infarction (MI), ischemic stroke and HHF. The secondary outcomes were the individual component of the primary composite endpoint. An encrypting procedure was used to link and continuously follow up all of the claims within the database belonging to the same patient. We followed each patient from the index date until December 31, 2019 which was the end of the cohort study. The longest follow up time was 10 years and the shortest follow-up time was 4 years. In Taiwan, ICD-10-CM codes were used to replace ICD-9-CM codes in the National Health Insurance from 2016, both ICD-9 and -10 codes were used to identify the outcome events.

### Statistical analysis

Continuous variables were presented as means ± standard deviations and categorical variables as numbers and percentages. The differences of clinical characteristics between groups were evaluated with absolute standardized mean difference (ASMD). An ASMD value < 0.1 indicates a negligible difference in potential confounders between two groups. To balance the confounding factors between the infection and control groups, we performed 1:1 propensity score matching using a greedy matching algorithm to generate matches with a caliper of 0.25 of the standard deviation of the logit of the propensity score. The matched variables included index year, age, gender, cormobidities (diabetes mellitus, hypertension, hyperlipidemia, coronary artery disease, peripheral artery disease, valvular heart disease, atrial fibrillation, ischemic stroke, intracerebral hemorrhage, chronic kidney disease, end-stage renal disease, chronic obstructive pulmonary disease, asthma, liver cirrhosis, peptic ulcers, gastrointestinal bleeding, malignancy, previous MI, previous percutaneous coronary intervention, previous coronary artery bypass graft, previous cardiac resynchronization therapy or implantable cardioverter-defibrillator) and medication history (angiotensin-converting enzyme inhibitors, angiotensin receptor blockers, angiotensin receptor-neprilysin inhibitor, beta-blocker, ivabradine, mineralocorticoid receptor antagonists, statin, diuretics, digoxin, hydralazine–isosorbide dinitrate, antiplatelets, anticoagulants, antiarrhythmics, oral hypoglycemic agents, insulin, non-steroidal anti-inflammatory drugs, steroid, colchicine). Multivariate Cox proportional hazards model was used in outcome assessment and to evaluate differences between infection and control groups. HR and 95% CI were calculated from the Cox models after adjusting for potential confounders. The Kaplan–Meier curve was generated for the primary outcome and the log-rank test was used to analyze statistic differences. Subgroup analyses were performed and the *p* values for interactions of the primary outcome with the same Cox proportional hazards model were calculated. To assess the influence of different infection on CV outcome, we performed another propensity score matching for patients with pneumonia or UTI and analyzed differences of primary outcome in multivariate Cox proportional hazards model. We performed several sensitivity analyses to reduced bias due to nonrandomized nature. The first is the competing risk analysis to regard mortality as a competing risk factor while reporting the differences of individual CV outcome. Second, we analyzed outcomes by fixed year after discharge. Finally, we added hypothetical unmeasured confounders into original survival regression by the R 4.2.1-package “obsSens” (R Foundation for Statistical Computing, Vienna, Austria)^15^. We examined the relationship of these unmeasured confounders assuming that the presence of different prevalence in the infection or control groups might affect the HRs for our primary outcome. The estimated trend of other potential residual confounders that may influence the observed results was calculated. All statistical tests in our study were 2-sided, and considered *p* value less than 0.05 was statistically significant. The SAS 9.4 for Windows (SAS Institute Inc., Cary, NC, USA) was used for all data analyses.

## Results

**Figure 1:**
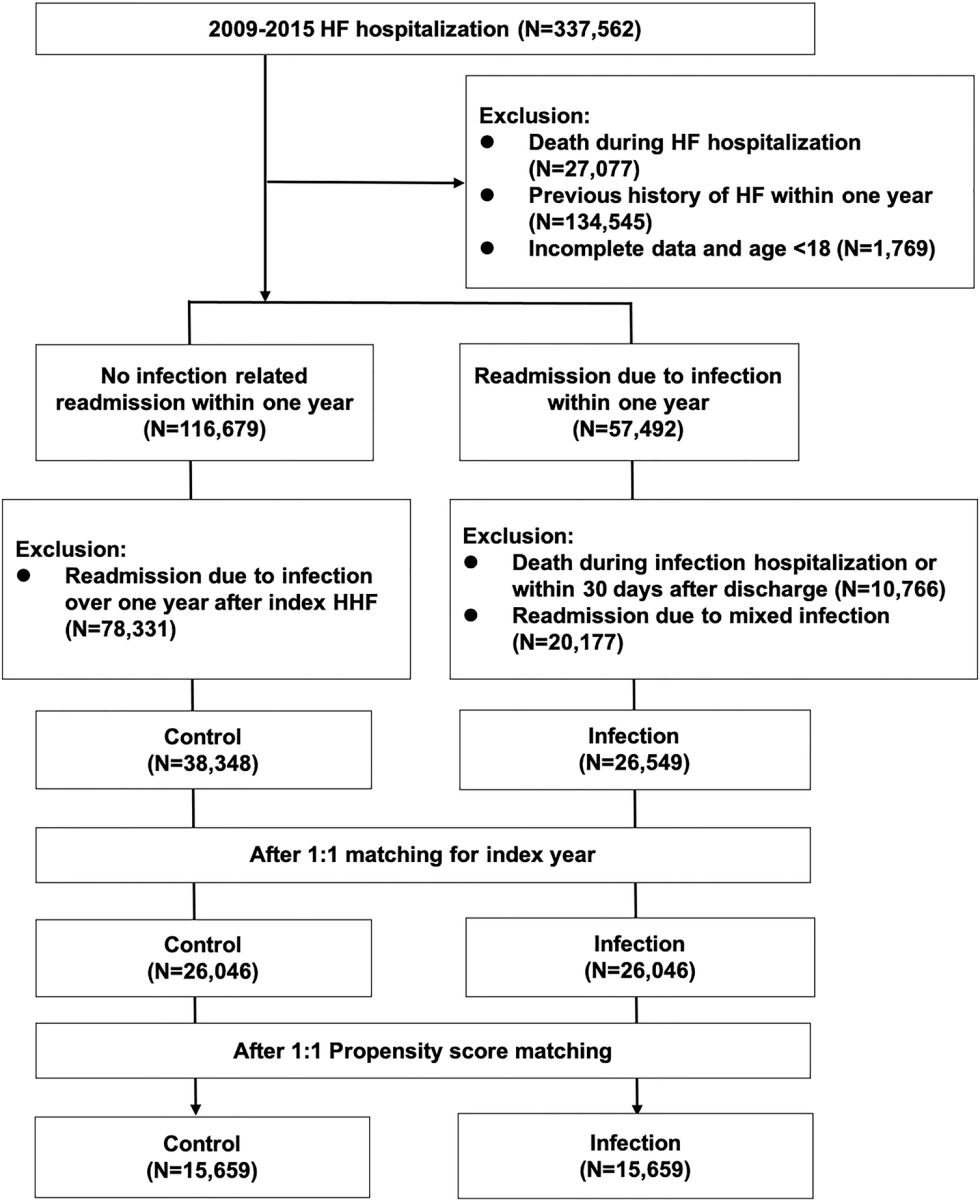
Flowchart of patient selection. *HF* heart failure.

Figure 1 shows the inclusion and exclusion of the study participants. From 2009 to 2015, 337,562 patients with their first HF admissions were identified from the NHIRD and 174,171 patients were enrolled in our study after exclusion. Among these patients, 57,492 (17.0%) were readmitted for infectious diseases within 1 year of HF discharge. Pneumonia (43.9%) was the most common type of infection, followed by UTI (38.2%), skin and soft tissue infections (9.8%), and others (8.2%). For infection group, we further excluded patients with mixed sites infections and those who died during hospitalization for infection or within 30 days after discharge. For control group without infection-related readmission within one year, we further excluded patients with any infection admission after one year of the index HF discharge. Overall, 26,549 patients in the infection group and 38,348 patients in the control group formed the basis of our primary analysis. We first matched for the index year of HF admission between groups. After the first matching, patients with incident infection were older and had more male and comorbidities than the control group (Table 1). After propensity score matching, the age, gender, clinical characteristics, morbidities and treatment were all well balanced between the 2 groups (n = 15,659 in each group).

**Table 1:** Patient characteristics at baseline and after propensity score matching.

	Propensity score matching							
	Before				After			
	Total (N = 52,092)	Control (N = 26,046)	Infection (N = 26,046)	ASMD	Total (N = 31,318)	Control (N = 15,659)	Infection (N = 15,659)	ASMD
Age	69.75 ± 15.58	64.64 ± 15.91	74.86 ± 13.42	0.695	71.71 ± 13.92	71.10 ± 13.21	72.31 ± 14.58	0.087
Male	28,163 (54.06)	15,401 (59.13)	12,762 (49.00)	0.204	16,290 (52.01)	8194 (52.33)	8096 (51.70)	0.013
Diabetes mellitus	22,295 (42.80)	8743 (33.57)	13,552 (52.03)	0.380	14,063 (44.90)	6813 (43.51)	7250 (46.30)	0.056
Hypertension	38,803 (74.49)	17,557 (67.41)	21,246 (81.57)	0.329	24,275 (77.51)	12,012 (76.71)	12,263 (78.31)	0.038
Hyperlipidemia	14,956 (28.71)	8508 (32.67)	6448 (24.76)	0.176	9089 (29.02)	4678 (29.87)	4411 (28.17)	0.038
Coronary artery disease	28,288 (54.30)	14,614 (56.11)	13,674 (52.50)	0.073	16,938 (54.08)	8480 (54.15)	8458 (54.01)	0.003
Previous MI	8395 (16.12)	4502 (17.28)	3893 (14.95)	0.064	4597 (14.68)	2276 (14.53)	2321 (14.82)	0.008
Previous PCI	7627 (14.64)	4493 (17.25)	3134 (12.03)	0.148	4073 (13.01)	2039 (13.02)	2034 (12.99)	0.001
Previous CABG	1670 (3.21)	961 (3.69)	709 (2.72)	0.055	1009 (3.22)	521 (3.33)	488 (3.12)	0.012
Previous CRT/ICD	144 (0.28)	83 (0.32)	61 (0.23)	0.016	82 (0.26)	42 (0.27)	40 (0.26)	0.003
Peripheral artery disease	1170 (2.25)	394 (1.51)	776 (2.98)	0.099	736 (2.35)	345 (2.20)	391 (2.50)	0.019
Valvular heart disease	10,028 (19.25)	5294 (20.33)	4734 (18.18)	0.055	6201 (19.80)	3164 (20.21)	3037 (19.39)	0.020
Atrial fibrillation	10,583 (20.32)	4996 (19.18)	5587 (21.45)	0.056	6511 (20.79)	3246 (20.73)	3265 (20.85)	0.003
Ischemic stroke	7611 (14.61)	2296 (8.82)	5315 (20.41)	0.333	4419 (14.11)	2009 (12.83)	2410 (15.39)	0.074
ICH	1402 (2.69)	294 (1.13)	1108 (4.25)	0.194	682 (2.18)	280 (1.79)	402 (2.57)	0.053
CKD	10,996 (21.11)	3700 (14.21)	7296 (28.01)	0.343	6890 (22.00)	3268 (20.87)	3622 (23.13)	0.055
ESRD	4579 (8.79)	1641 (6.30)	2938 (11.28)	0.177	3055 (9.75)	1488 (9.50)	1567 (10.01)	0.017
COPD	14,530 (27.89)	4175 (16.03)	10,355 (39.76)	0.549	8446 (26.97)	3827 (24.44)	4619 (29.50)	0.114
Asthma	5146 (9.88)	1580 (6.07)	3566 (13.69)	0.258	3120 (9.96)	1425 (9.10)	1695 (10.82)	0.058
Liver cirrhosis	1628 (3.13)	537 (2.06)	1091 (4.19)	0.123	973 (3.11)	452 (2.89)	521 (3.33)	0.025
Peptic ulcers	11,647 (22.36)	4148 (15.93)	7499 (28.79)	0.313	7005 (22.37)	3359 (21.45)	3646 (23.28)	0.044
Gastrointestinal bleeding	10,413 (19.99)	3603 (13.83)	6810 (26.15)	0.312	6200 (19.80)	2920 (18.65)	3280 (20.95)	0.058
Malignancy	38,867 (74.61)	17,385 (66.75)	21,482 (82.48)	0.368	23,967 (76.53)	11,798 (75.34)	12,169 (77.71)	0.056
Medication history								
ACEI/ARB/ARNI	25,877 (49.68)	13,646 (52.39)	12,231 (46.96)	0.109	15,700 (50.13)	7972 (50.91)	7728 (49.35)	0.031
Beta-blocker	21,847 (41.94)	12,263 (47.08)	9584 (36.80)	0.210	12,833 (40.98)	6581 (42.03)	6252 (39.93)	0.043
Ivabradine	60 (0.12)	32 (0.12)	28 (0.11)	0.005	35 (0.11)	19 (0.12)	16 (0.10)	0.006
MRA	8967 (17.21)	3969 (15.24)	4998 (19.19)	0.105	5358 (17.11)	2576 (16.45)	2782 (17.77)	0.035
Statin	12,876 (24.72)	7818 (30.02)	5058 (19.42)	0.248	7471 (23.86)	3883 (24.80)	3588 (22.91)	0.044
Diuretics	22,195 (42.61)	8754 (33.61)	13,441 (51.60)	0.370	13,886 (44.34)	6712 (42.86)	7174 (45.81)	0.059
Digoxin	6221 (11.94)	2875 (11.04)	3346 (12.85)	0.056	3848 (12.29)	1906 (12.17)	1942 (12.40)	0.007
H-ISDN	1050 (2.02)	306 (1.17)	744 (2.86)	0.120	588 (1.88)	271 (1.73)	317 (2.02)	0.022
Antiplatelet	24,805 (47.62)	12,737 (48.90)	12,068 (46.33)	0.051	14,837 (47.38)	7427 (47.43)	7410 (47.32)	0.002
Anticoagulant	4978 (9.56)	2682 (10.30)	2296 (8.82)	0.050	3144 (10.04)	1641 (10.48)	1503 (9.60)	0.029
Antiarrhythmic	5818 (11.17)	2578 (9.90)	3240 (12.44)	0.081	3469 (11.08)	1710 (10.92)	1759 (11.23)	0.010
OHA	14,164 (27.19)	5921 (22.73)	8243 (31.65)	0.201	9067 (28.95)	4442 (28.37)	4625 (29.54)	0.026
Insulin	7408 (14.22)	1888 (7.25)	5520 (21.19)	0.408	4024 (12.85)	1757 (11.22)	2267 (14.48)	0.097
NSAID	13,371 (25.67)	6533 (25.08)	6838 (26.25)	0.027	8407 (26.84)	4202 (26.83)	4205 (26.85)	0.000
Steroid	10,298 (19.77)	3052 (11.72)	7246 (27.82)	0.413	5758 (18.39)	2596 (16.58)	3162 (20.19)	0.093
Colchicine	3082 (5.92)	1323 (5.08)	1759 (6.75)	0.071	1876 (5.99)	914 (5.84)	962 (6.14)	0.013

*ASMD* absolute standardized mean difference, *MI* myocardial infarction, *PCI* percutaneous coronary intervention, *CABG* coronary artery bypass graft, *CRT* cardiac resynchronization therapy, *ICD* implantable cardioverter-defibrillator, *ICH* intracerebral hemorrhage, *CKD* chronic kidney disease, *ESRD* end-stage renal disease, *COPD* chronic obstructive pulmonary disease, *ACEI* angiotensin-converting enzyme inhibitors, *ARB* angiotensin receptor blockers, *ARNI* angiotensin receptor-neprilysin inhibitor, *MRA* mineralocorticoid receptor antagonists, *H-ISDN* hydralazine–isosorbide dinitrate, *OHA* oral hypoglycemic agents, *NSAID* non-steroidal anti-inflammatory drugs.

**Figure 2:**
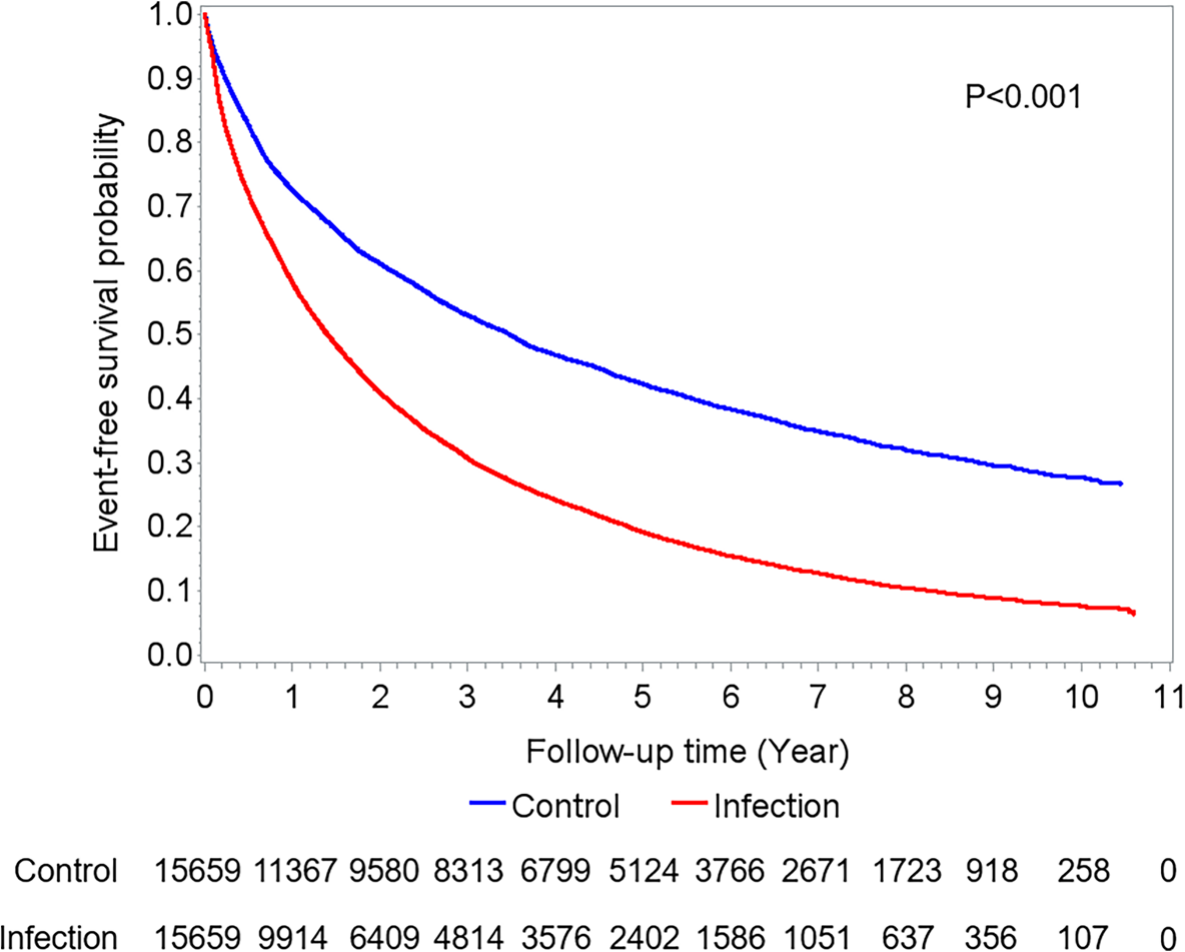
Kaplan–Meier curve for the primary composite endpoint. The blue and orange lines represent event-free survival in the infection and control groups, respectively. The negative impact of infection is not only present in the acute or subacute phase, but also persists during long term follow-up.

After a mean follow-up time of 4.29 ± 2.92 years, 86.19% patients with a history of infection-related admission and 63.63% patients in the control group met the primary composite endpoint. Multivariate Cox proportional hazards analysis showed the infection group had a higher risk in the primary composite endpoint (adjusted HR 1.760, 95% CI 1.714–1.807, *p* < 0.001) and all its components (Table 2). The adjusted HRs were 1.587 for the all-cause death (95% CI 1.540–1.636, *p* < 0.001), 1.993 for HHF (95% CI 1.922–2.066, *p* < 0.001), 1.332 for MI (95% CI 1.224- 1.450, *p* < 0.001), and 1.769 for ischemic stroke (95% CI 1.664–1.882, *p* < 0.001). The risks of all components of the primary outcome were persistently higher in the infection group after adjusting for mortality as a competing risk or by fixed-year model (Supplementary Table S2 and S3). The Kaplan Meier curve showed a lower event-free survival in the infection group. The difference started early and persisted until the end of follow-up (Fig. 2). Among the primary composite endpoint, death was the most common event, with 69.44% of patients in the infection group and 45.85% of patients in the control group eventually died during the long-term follow-up. While HHF was the second most common event (49.66% in the infection group vs. 31.90% in the control group) and it was the earliest outcome event in both the infection group (time to event: 523.73 ± 595.40 days) and the control group (time to event: 693.68 ± 733.37 days) (Supplementary Table S4). There was an impact of different site infection on primary outcomes. We matched the propensity scores in patients with pneumonia and UTI (Supplementary Table S5) and compared all endpoints in a Cox proportional hazards model (Table 3). The risk of the primary composite endpoint was significantly higher in patients with pneumonia than UTI (HR 1.140, 95% CI 1.104–1.178, *p* < 0.001). The risks of all-cause mortality (HR 1.136, 95% CI 1.096–1.177, *p* < 0.001) and HHF (HR 1.141, 95% CI 1.091–1.192, *p* < 0.001) were higher in patients with pneumonia, but not for MI (HR 1.033, 95% CI 0.923–1.155, *p* = 0.574) or ischemic stroke (HR 0.994, 95% CI 0.921–1.074, *p* = 0.886).

**Table 2:** Clinical outcome of infection versus control group.

	Total (N = 31,318)	Control (Ref.) (N = 15,659)	Infection (N = 15,659)	Crude HR (95% CI)	*p* value	Adjusted HR (95% CI)	*p* value
Primary composite endpoint	23,460 (74.91)	9964 (63.63)	13,496 (86.19)	1.885 (1.837–1.935)	< 0.001	1.760 (1.714–1.807)	< 0.001
Mortality	18,053 (57.64)	7180 (45.85)	10,873 (69.44)	1.796 (1.743–1.851)	< 0.001	1.587 (1.540–1.636)	< 0.001
HHF	12,773 (40.78)	4996 (31.90)	7777 (49.66)	2.054 (1.981–2.128)	< 0.001	1.993 (1.922–2.066)	< 0.001
MI	2244 (7.17)	1000 (6.39)	1244 (7.94)	1.470 (1.352–1.598)	< 0.001	1.332 (1.224–1.450)	< 0.001
Ischemic stroke	4353 (13.90)	1746 (11.15)	2607 (16.65)	1.872 (1.761–1.989)	< 0.001	1.769 (1.664–1.882)	< 0.001

Model was adjusted for age, sex, comorbidities, procedure history, medication history.

*HR* hazard ratio, *HHF* hospitalization for heart failure, *MI* myocardial infarction.

**Table 3:** Clinical outcome of pneumonia versus urinary tract infection group.

	Total (N = 16,114)	Pneumonia (N = 8057)	Urinary Tract Infection (Ref.) (N = 8057)	Crude HR (95% CI)	*p* value	Adjusted HR (95% CI)	*p* value
Primary composite endpoint	14,601 (74.91)	7377 (91.56)	7224 (89.66)	1.154 (1.118–1.193)	< 0.001	1.140 (1.104–1.178)	< 0.001
Mortality	12,549 (57.64)	6382 (79.21)	6167 (76.54)	1.128 (1.089–1.168)	< 0.001	1.136 (1.096–1.177)	< 0.001
HHF	7992 (40.78)	4060 (59.39)	3932 (48.80)	1.164 (1.114–1.217)	< 0.001	1.141 (1.091–1.192)	< 0.001
MI	1259 (7.17)	634 (7.87)	625 (7.76)	1.103 (0.988–1.232)	0.081	1.033 (0.923–1.155)	0.574
Ischemic stroke	2651 (13.90)	1269 (15.75)	1382 (17.15)	1.000 (0.927–1.079)	1.000	0.994 (0.921–1.074)	0.886

Model was adjusted for age, sex, comorbidities, procedure history, medication history.

*HR* hazard ratio, *HHF* hospitalization for heart failure, *MI* myocardial infarction.

**Figure 3:**
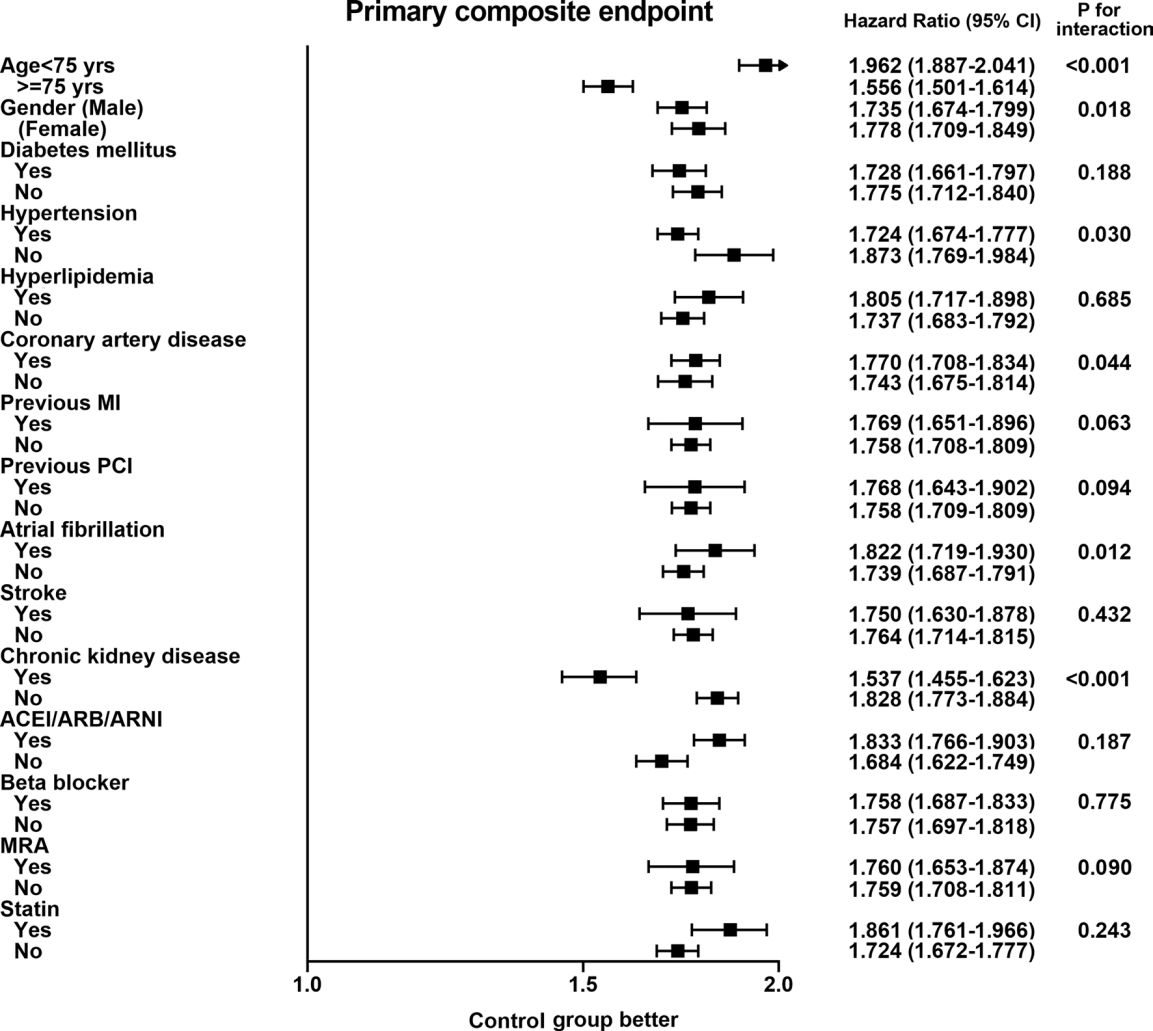
Forest plot of subgroup analysis. Subgroup analysis showed the risk was consistently higher in the infection group than the controls in all subgroups. P for interaction test showed the impact of infection may vary by age, gender, and in patients with a history of hypertension, atrial fibrillation, and chronic kidney disease. *MI* myocardial infarction, *PCI* percutaneous coronary intervention, *ACEI* angiotensin-converting enzyme inhibitors, *ARB* angiotensin receptor blockers, *ARNI* angiotensin receptor-neprilysin inhibitor, *MRA* mineralocorticoid receptor antagonists.

In the subgroup analysis (Fig. 3), a consistent pattern of worse prognosis after infection in all subgroups. The impact of infection on long-term outcome was much higher in patients younger than 75 years and without a history of CKD (*p* for interaction < 0.001). After adding the hypothetical unmeasured confounders to the model, the risk for the infection group remained higher than the risk for the control group in most cases. Supplementary Figure S1 shows the trend estimates for the infection group in the covariate-adjusted Cox proportional hazards model. When the prevalence of unmeasured confounders is higher in the control group and lower in the infected group, the HR of infection will be greater. Assuming a 1.0 prevalence of unmeasured confounders in the control group and a 0.0 prevalence of the confounders in the infected group, the HR may increase to fourfold.

## Discussion

The results of this long term follow up study showed that 17% HF patients were likely to be readmitted due to acute infection within one year after their first HF admissions. Infection-related readmission not only elevated the risk of subsequent mortality and HHF, but also increased acute attack of MI and ischemic stroke. The worse long-term CV prognosis was observed persistently over a 10-year follow up than those without the infection episode.

While hospitalization for HF has received increasing attention from clinicians and public health researchers, a recent study has shown that although the proportion of hospitalizations due to HF or other cardiovascular diseases has remained stable, there has been a notable increase in the proportion of non-cardiac hospitalizations, especially those related to infection^16^. However, the impact of infection-related hospitalization for patients with HF remains largely unknown.

Shen et al. reported that the risk of mortality in HF patients increased to about fourfold higher after an episode of pneumonia than those without infection and the risk was extremely high in the first month after pneumonia^11^. Drozd et al. also reported that infection in HFrEF was associated with an age-sex adjusted 3.6-fold greater risk of death during the infection-related hospitalization than hospitalization for other reasons^10^. In our study, we excluded patients who died during acute infection or within 30 days of discharge because the purpose of this study was to observe a long-term effect of infection. We hypothesized that the negative influence of early infection is not only present in the acute or subacute phase, but can persistently lead to long-term sequelae even if the patients recover from the infection episode completely. We showed that even we excluded the mortality during the infection-related admission and 30 days after discharge, the subsequent risk of mortality or HHF were still high. The event-free survival curves continued to diverge in the infection group compared to the control group and persisted broadly parallel over to 10 years follow up.

Despite the progress of HF therapies, we found the long-term prognosis after initial HF admission still remained poor and infection episode worsened the clinical outcomes. In our study, after a mean follow-up of 4.2 years, the survival rate in the control group was only 54.15%. After hospitalization for infection, the prognosis became worse with the survival rate dropping to 30.56%. Our findings were similar to a prospective cohort study in the United Kingdom (UK) that the 4-year survival rate after infection-related hospitalization was approximately 30% in HFrEF^10^. Infection is well known as a major predisposing factor of acute decompensated HF. Acute infections may increase myocardial demand and activate the renin–angiotensin–aldosterone system (RASS) and sympathetic nervous system^17,18^. Sepsis may cause direct cardiomyocyte injury which is induced by toxins, complement and damage-associated molecular patterns (DAMPs). Further downregulation of β-adrenergic receptors and post-receptor signaling pathways may also inhibit left ventricle contractility^19^. Hypoxemia during pneumonia or respiratory infection may lead to pulmonary artery constriction and increased right ventricular afterload^20^. Furthermore, a hypercoagulable state may persist after an infection episode and lead to atherosclerotic plaque instability^21,22^. As a result, plenty of observational studies have shown a higher risk of MI after influenza infection^23,24^. Despite the fact that influenza or pneumococcal vaccination is widely recommended by guidelines, the evidence of efficacy remains insufficient. In the PARADIGM-HF trial, influenza vaccination was associated with a reduced risk of all-cause mortality in HFrEF^25^. However, meta-analyses from 6 different cohort studies showed no statistically significant effect of influenza vaccination on CV mortality or all-cause hospitalization^26^. A recent randomized double-blind clinical trial showed that influenza vaccination after MI or in patients with high-risk coronary artery disease reduced the risk of composite CV endpoints and CV death^27^. But another randomized trial showed influenza vaccination could not achieve a statistically significant differences of the primary endpoints in HF patients^28^.

Musher et al. reported that patients with pneumococcal pneumonia were at increased risk for concurrent acute cardiac events, including MI, severe arrhythmias, and worsening HF^29^. A prospective registry of patients with community-acquired pneumonia also found that 11.9% of patients with pneumonia has incident HF compared to 7.4% of controls (adjusted HR 1.61, 95% CI 1.44–1.81) over nearly 10 years of follow-up^30^. Other community-based cohort studies have also reported that pneumonia may increase the risk of major cardiovascular events in the long term^31^. Our study also addresses another interesting issue, that even urinary tract infections may also lead to poorer CV outcomes. According to our statistics, the risk of urinary tract infections was slightly lower than that of pneumonia. However, 89.66% of patients with urinary tract infections and 91.56% of patients with pneumonia met the primary composite endpoint at long-term follow-up, which is not very different at the numerical level. This finding has some similarity to the study by Shen et al. In the post-hoc studies of the Paradigm-HF and Paragon-HF trials, they also mentioned that patients were at higher risk after UTI, although the elevated risk was substantially lower than in pneumonia^11^.

## Strengths and limitations

Our study provides a large-scale and real-world evidence of the long-term negative impact of infection on all CV endpoints, including mortality, HHF, MI and stroke in patients with HF. The results were consistent across all subgroups, including age, sex, comorbidities, and guideline-guided medication use. There were several strengths in our study. First, most previous studies have largely focused on short-term influence of infection, but our study provided long-term follow-up results and demonstrated that the impact could persisted up to 10 years. Second, previous studies have mainly reported the increased risk of mortality after infection episode, but we showed that infection actually affected all important CV endpoints, such as HHF, MI and stroke in those who survived after infection. Third, our study had a much larger number of HF patients than before and the large case number provided an opportunity to detect the MI and stroke risk after infection in the long term follow up. Fourth, we pointed out that not only pneumonia may affect the outcome, but UTI may be evil. A novel strategy of infection prevention may need to be developed for HF patients. Our study also has limitations. First, our study was a nonrandomized retrospective cohort study. Although we performed propensity score matching, that reflect the balance of measured confounders, potentially unmeasured confounders and selection bias may not be entirely eliminated^32,33^. To increase reliability, we incorporated hypothetical unmeasured confounders in our sensitivity analysis by simulating potential unmeasured variables that could affect the treatment effect estimate. However, if the prevalence of unmeasured confounders are strongly associated with a particular group, the effect may not be adjusted, and it is an inherent limitation of retrospective studies. Second, we did not know whether infection affects differently in patients with HFrEF, mildly reduced ejection fraction (HFmrEF), or HFpEF because the detailed echocardiographic measurements were not available in our database. However, previous studies may give us clues that the elevated risk of HHF and CV death after pneumonia is fairly consistent in either the HFrEF or HFpEF cohort^11^. Finally, the bacterial etiology of the infectious events were not known. Etiologies other than bacteria of these infectious events could occur. The influence of different etiologies of infection on the clinical outcomes was not able to be evaluated.

## Conclusions

We found persistent adverse CV prognosis even after HF patients recovered from acute infection. Patients with history of infection had a twofold higher risk of HHF and a 1.5-fold higher risk of mortality than those without infection. Further studies are warranted to understand the underlying mechanisms and to provide preventive strategies.

## Supplementary Information

Supplementary Information.

## Data Availability

The datasets used and/or analysed during the current study available from the corresponding author on reasonable request.

## Abbreviations

HF: Heart failure
MACE: Major adverse cardiovascular events
MI: Myocardial infarction
HHF: Hospitalization for HF
HFrEF: HF with reduced ejection fraction
HFpEF: HF with preserved ejection fraction
HR: Hazard ratio
CV: Cardiovascular
UTI: Urinary tract infection
