# Supplementary material for: Impact of infection-related admission in patients with heart failure: a 10 years national cohort study
Source: Sci Rep. 2023 Apr 28;13:6941. doi: 10.1038/s41598-023-34028-8 (PMC10147930; doi:10.1038/s41598-023-34028-8)
Supplement: Supplementary file 1 — Supplementary Information. [file 41598_2023_34028_MOESM1_ESM.docx]

**Supplementary appendix**

**Contents**

Supplementary Table S1. ICD codes

Supplementary Table S2. Competing risk model for different cardiovascular outcome

Supplementary Table S3. Fixed-year model for outcome assessment

Supplementary Table S4. Time to event of infection vs. control

Supplementary Table S5. Baseline characteristics and after propensity score matching for pneumonia and UTI comparison

Supplementary Table S6. Time to event of pneumonia vs. UTI

Supplementary Table S7. Outcome assessment adjusted by length of infection admission, infection vs. control

Supplementary Figure S1. Sensitivity analysis of unmeasured confounders

**Supplementary table S1. ICD codes**

| Disease | ICD-9-CM Codes | ICD-10-CM Codes |
| --- | --- | --- |
| Pneumonia | 480-486 | J12-J18, J851 |
| UTI | 595.0, 599.0, 590.1, 590.2, 590.9 | N30.0, N39.0, N10, N15.1 |
| SSTI | 376.01, 680-682, 684, 686 | L00-L03, L08, M72.6, H05.01 |
| Other infection  (BTI, IAI, septic arthritis, osteomyelitis, IE) | 575.0, 572.0, 574.3, 574.6, 576.1, 540, 569.5, 566, 567, 730.0, 711, 421.0 | K81.0, K83.0, K80.3, K80.4, K75.0, K35, K61, K63.0, K67, K65, K68.1, M00, M01, M86, I33 |
| Sepsis/Bacteremia | 790.7, 995.91, 995.92 | R78.81, A41.9, R65.2 |
| Congestive heart failure | 428 | I50.1, I50.20, I50.21, I50.22, I50.23, I50.30, I50.31, I50.32, I50.33, I50.40, I50.41, I50.42, I50.43, I50.9 |
| Myocardial infarction | 410 | I21,I22,I23 |
| Coronary artery disease | 410, 411, 412, 413, 414 | I21-I25 |
| Valvular heart disease | 394, 395, 396, 397,  424.0-424.3,746.3-746.6 | I34-I37, I05-08, Q23.0-Q23.3, Q23.8.Q23.9 |
| Prior CABG | 36.1, 36.2 | 0210-0213 |
| Prior PCI | 00.66, 36.06, 36.07 | 02703, 02713, 02723, 02733 |
| Stroke | 433, 434, 436 | I63, I65, I66, I67, I68, I69 |
| ICH | 430, 431, 432, 852, 853 | I60, I61, I62 |
| Peripheral artery disease | 440.2, 440.3, 440.4, 443.9 | I659,I6300,I6310,I6320,I6329, I73.9, I70.2, I70.3, I70.4, I70.5, I70.6, I70.7, I70.8 |
| Hypertension | 401, 402, 403, 404, 405 | I10-I16 |
| Diabetes mellitus | 250 | E10, E11 |
| Hyperlipidemia | 272.0, 272.1, 272.2, 272.3, 272.4, 272.9 | E78 |
| Chronic obstructive lung disease | 491, 492, 494, 495, 496 | J41.0, J41.1, J41.8, J42, J43.0,  J43.1, J43.2, J43.8, J43.9, J44.0,  J44.1, J44.9, J47.0, J47.1, J47.9,  J67.0, J67.1, J67.2, J67.3, J67.4,  J67.5, J67.6, J67.7, J67.8, J67.9 |
| Asthma | 493 | J44.0, J44.1, J44.9, J45.20, J45.21, J45.22, J45.30, J45.31, J45.32, J45.40, J45.41, J45.42, J45.50, J45.51, J45.52, J45.901, J45.902, J45.909,J45.991,J45.998 |
| Peptic ulcers | 531-534, 530.2, V12.71 | K25, K26, K27 |
| Gastrointestinal bleeding | 456.0, 456.2, 455.2, 455.5, 455.8, 530.7, 530.82, 531.0-531.6, 532.0-532.6, 533.0-533.6, 534.0-534.6, 535.0-535.6 537.83, 562.02, 562.03, 562.12 562.13 568.81, 569.3, 569.85, 578.0, 578.1, 578.9 | K25.0, K26.0, K27.0, K28.0, K29.0 |
| Liver cirrhosis | 571.2; 571.5; 571.6 | K70.3; K74; K74.60 |
| Malignancy | 140.0-208.9 | C |
| Atrial fibrillation | 427.31、427.32 | I48 |
| Chronic kidney disease | 580-589 | I12, I13, N00, N01, N02, N03, N04, N05, N07, N11, N14, N17, N18, N19, Q61 |
| ESRD | 585 | N18.5; N18.6 |

UTI= urinary tract infection; SSTI= skin and soft tissue infection; BTI= biliary tract infection; IAI= intra-abdominal infection; IE= infectious endocarditis; PCI= percutaneous coronary intervention; CABG= coronary artery bypass graft; ICH= intracerebral hemorrhage; ESRD= end-stage renal disease

| **Supplementary table S2. Competing risk model for different cardiovascular outcome** | | | | | | | |
| --- | --- | --- | --- | --- | --- | --- | --- |
|  | Total  N=31318 | Control (Ref.)  N=15659 | Infection  N=15659 | Crude SHR  (95%CI) | *p* value | Adjusted SHR (95%CI) | *p* value |
| HHF | 12773 (40.78) | 4996 (31.90) | 7777 (49.66) | 1.911 (1.845-1.979) | <0.001 | 1.960 (1.891- 2.033) | <0.001 |
| MI | 2244 (7.17) | 1000 (6.39) | 1244 (7.94) | 1.271 (1.170-1.381) | <0.001 | 1.228 (1.128-1.337) | <0.001 |
| Ischemic stroke | 4353 (13.90) | 1746 (11.15) | 2607 (16.65) | 1.576 (1.484-1.674) | <0.001 | 1.569 (1.475-1.668) | <0.001 |
| Model was adjusted for age, sex, comorbidities, procedure history, medication history.  SHR=Subdistribution hazard ratio. | | | | | | | |

**Supplementary table S3. Fixed-year model for outcome assessment**

| 1Y |  |  |  |  |  |  |  |  |  |  |  |
| --- | --- | --- | --- | --- | --- | --- | --- | --- | --- | --- | --- |
|  | Total | Control (Ref.) | Infection | Crude | | | | Adjusted | | | |
|  | N=31318 | N=15659 | N=15659 | HR | 95% CI | | P | HR | 95% CI | | P |
| Composite endpoint | 10852 (34.65) | 4297 (27.44) | 6555 (41.86) | 1.690 | 1.626 | 1.756 | <0.001 | 1.538 | 1.479 | 1.599 | <0.001 |
| Mortality | 5540 (17.69) | 2415 (15.42) | 3125 (19.96) | 1.326 | 1.257 | 1.398 | <0.001 | 1.111 | 1.053 | 1.173 | <0.001 |
| HHF | 6340 (20.24) | 2307 (14.73) | 4033 (25.76) | 1.916 | 1.820 | 2.016 | <0.001 | 1.815 | 1.723 | 1.911 | <0.001 |
| MI | 788 (2.52) | 372 (2.38) | 416 (2.66) | 1.150 | 1.000 | 1.322 | 0.051 | 1.012 | 0.878 | 1.165 | 0.874 |
| Stroke | 1270 (4.06) | 509 (3.25) | 761 (4.86) | 1.549 | 1.385 | 1.733 | <0.001 | 1.422 | 1.270 | 1.593 | <0.001 |
|  |  |  |  |  |  |  |  |  |  |  |  |
|  |  |  |  |  |  |  |  |  |  |  |  |
| 2Y |  |  |  |  |  |  |  |  |  |  |  |
|  | Total | Control (Ref.) | Infection | Crude | | | | Adjusted | | | |
|  | N=31318 | N=15659 | N=15659 | HR | 95% CI | | P | HR | 95% CI | | P |
| Composite endpoint | 15339 (48.98) | 6085 (38.86) | 9254 (59.10) | 1.790 | 1.733 | 1.849 | <0.001 | 1.638 | 1.585 | 1.693 | <0.001 |
| Mortality | 8963 (28.62) | 3787 (24.18) | 5176 (33.05) | 1.435 | 1.376 | 1.496 | <0.001 | 1.218 | 1.167 | 1.271 | <0.001 |
| HHF | 8797 (28.09) | 3181 (20.31) | 5616 (35.86) | 2.038 | 1.951 | 2.129 | <0.001 | 1.947 | 1.863 | 2.035 | <0.001 |
| MI | 1230 (3.93) | 564 (3.60) | 666 (4.25) | 1.245 | 1.113 | 1.392 | <0.001 | 1.118 | 0.998 | 1.252 | 0.055 |
| Stroke | 2133 (6.81) | 818 (5.22) | 1315 (8.40) | 1.726 | 1.582 | 1.884 | <0.001 | 1.602 | 1.466 | 1.750 | <0.001 |
|  |  |  |  |  |  |  |  |  |  |  |  |
|  |  |  |  |  |  |  |  |  |  |  |  |
| 3Y |  |  |  |  |  |  |  |  |  |  |  |
|  | Total | Control (Ref.) | Infection | Crude | | | | Adjusted | | | |
|  | N=31318 | N=15659 | N=15659 | HR | 95% CI | | P | HR | 95% CI | | P |
| Composite endpoint | 18199 (58.11) | 7350 (46.94) | 10849 (69.28) | 1.825 | 1.772 | 1.880 | <0.001 | 1.679 | 1.630 | 1.731 | <0.001 |
| Mortality | 11644 (37.18) | 4830 (30.84) | 6814 (43.51) | 1.521 | 1.466 | 1.578 | <0.001 | 1.305 | 1.257 | 1.355 | <0.001 |
| HHF | 10270 (32.79) | 3780 (24.14) | 6490 (41.45) | 2.064 | 1.983 | 2.149 | <0.001 | 1.983 | 1.904 | 2.065 | <0.001 |
| MI | 1553 (4.96) | 710 (4.53) | 843 (5.38) | 1.284 | 1.162 | 1.419 | <0.001 | 1.150 | 1.039 | 1.272 | 0.007 |
| Stroke | 2792 (8.92) | 1076 (6.87) | 1716 (10.96) | 1.773 | 1.643 | 1.914 | <0.001 | 1.659 | 1.536 | 1.792 | <0.001 |

**Supplementary table S4. Time to event of infection vs. control**

|  | Time to event, days (mean±SD) | | |
| --- | --- | --- | --- |
|  | ALL | No infection | Infection |
| Mortality | 733.99 ± 730.32 | 763.81 ± 745.48 | 708.75 ± 716.35 |
| HHF | 589.25 ± 657.25 | 693.68 ± 733.37 | 523.73 ± 595.40 |
| MI | 750.46 ± 741.03 | 781.45 ± 778.90 | 719.39 ± 700.70 |
| Stroke | 899.49 ± 784.08 | 963.22 ± 812.97 | 851.75 ± 758.51 |
| Primary composite endpoint | 679.63 ± 708.37 | 751.38 ± 751.97 | 626.65 ± 669.49 |

| **Supplementary table S5. Baseline characteristics and after propensity score matching for pneumonia and UTI comparison** | | | | | | | | | | | | | | |  | |  |
| --- | --- | --- | --- | --- | --- | --- | --- | --- | --- | --- | --- | --- | --- | --- | --- | --- | --- |
|  | Propensity Score Matching | | | | | | | | | | | | | | | | |
|  | Before | | | | | | |  | After | | | | | | | | |
| Variables | Total | | Pneumonia | | Urinary Tract Infection | | ASMD |  | Total | | Pneumonia | | Urinary Tract Infection | | | ASMD | |
|  | N=21356 | | N=11405 | | N=9951 | |  |  | N=16114 | | N=8057 | | N=8057 | | |  |  |
| Age | 76.12±12.72 | | 75.37±13.33 | | 76.98±11.93 | | 0.128 |  | 76.53 ± 12.49 | | 76.30 ± 12.76 | | 76.76 ± 12.21 | | | 0.037 | |
| Male | 10056 | (47.09) | 7035 | (61.68) | 3021 | (30.36) | 0.662 |  | 6806 | (42.24) | 3796 | (47.11) | 3010 | (37.36) | | 0.199 | |
| Diabetes mellitus | 11045 | (51.72) | 5373 | (47.11) | 5672 | (57.00) | 0.199 |  | 8475 | (52.59) | 4145 | (51.45) | 4330 | (53.74) | | 0.046 | |
| Hypertension | 17462 | (81.77) | 9107 | (79.85) | 8355 | (83.96) | 0.107 |  | 13298 | (82.52) | 6624 | (82.21) | 6674 | (82.83) | | 0.016 | |
| Hyperlipidemia | 5122 | (23.98) | 2600 | (22.80) | 2522 | (25.34) | 0.060 |  | 3845 | (23.86) | 1881 | (23.35) | 1964 | (24.38) | | 0.024 | |
| Coronary artery disease | 11170 | (52.30) | 6031 | (52.88) | 5139 | (51.64) | 0.025 |  | 8384 | (52.03) | 4215 | (52.31) | 4169 | (51.74) | | 0.011 | |
| History of MI | 3218 | (15.09) | 1780 | (15.61) | 1438 | (14.45) | 0.032 |  | 2407 | (14.94) | 1229 | (15.25) | 1178 | (14.62) | | 0.018 | |
| Prior PCI | 2486 | (11.64) | 1421 | (12.46) | 1065 | (10.70) | 0.055 |  | 1784 | (11.07) | 903 | (11.21) | 881 | (10.93) | | 0.009 | |
| Prior CABG | 516 | (2.42) | 293 | (2.57) | 223 | (2.24) | 0.021 |  | 397 | (2.46) | 207 | (2.57) | 190 | (2.36) | | 0.014 | |
| Received CRT/ICD | 44 | (0.21) | 26 | (0.23) | 18 | (0.18) | 0.010 |  | 28 | (0.17) | 13 | (0.16) | 15 | (0.19) | | 0.006 | |
| Peripheral artery disease | 556 | (2.60) | 280 | (2.46) | 276 | (2.77) | 0.020 |  | 425 | (2.64) | 212 | (2.63) | 213 | (2.64) | | 0.001 | |
| Valvular heart disease | 3825 | (17.91) | 2195 | (19.25) | 1630 | (16.38) | 0.075 |  | 2856 | (17.72) | 1443 | (17.91) | 1413 | (17.54) | | 0.010 | |
| Atrial fibrillation | 4695 | (21.98) | 2625 | (23.02) | 2070 | (20.80) | 0.054 |  | 3500 | (21.72) | 1765 | (21.91) | 1735 | (21.53) | | 0.009 | |
| Ischemic stroke | 4667 | (21.85) | 2202 | (19.31) | 2465 | (24.77) | 0.132 |  | 3615 | (22.43) | 1789 | (22.20) | 1826 | (22.66) | | 0.011 | |
| ICH | 1001 | (4.69) | 499 | (4.38) | 502 | (5.04) | 0.032 |  | 800 | (4.96) | 411 | (5.10) | 389 | (4.83) | | 0.013 | |
| CKD | 5778 | (27.06) | 3067 | (26.89) | 2711 | (27.24) | 0.008 |  | 4322 | (26.82) | 2132 | (26.46) | 2190 | (27.18) | | 0.016 | |
| ESRD | 2091 | (9.79) | 1371 | (12.02) | 720 | (7.24) | 0.163 |  | 1465 | (9.09) | 773 | (9.59) | 692 | (8.59) | | 0.035 | |
| COPD | 9132 | (42.76) | 5903 | (51.76) | 3229 | (32.45) | 0.399 |  | 6400 | (39.72) | 3349 | (41.57) | 3051 | (37.87) | | 0.076 | |
| Asthma | 3174 | (14.86) | 2127 | (18.65) | 1047 | (10.52) | 0.232 |  | 2183 | (13.55) | 1171 | (14.53) | 1012 | (12.56) | | 0.058 | |
| Liver cirrhosis | 756 | (3.54) | 370 | (3.24) | 386 | (3.88) | 0.034 |  | 575 | (3.57) | 277 | (3.44) | 298 | (3.70) | | 0.014 | |
| Peptic ulcers | 6134 | (28.72) | 3126 | (27.41) | 3008 | (30.23) | 0.062 |  | 4635 | (28.76) | 2293 | (28.46) | 2342 | (29.07) | | 0.013 | |
| Gastrointestinal bleeding | 5669 | (26.54) | 2847 | (24.96) | 2822 | (28.36) | 0.077 |  | 4351 | (27.00) | 2167 | (26.90) | 2184 | (27.11) | | 0.005 | |
| Malignancy | 17581 | (82.32) | 9190 | (80.58) | 8391 | (84.32) | 0.099 |  | 13302 | (82.55) | 6607 | (82.00) | 6695 | (83.10) | | 0.029 | |
| **Medication history** |  |  |  |  |  |  |  |  |  |  |  |  |  |  | |  | |
| ACEI/ARB/ARNI | 9891 | (46.31) | 5189 | (45.5) | 4702 | (47.25) | 0.035 |  | 7422 | (46.06) | 3684 | (45.72) | 3738 | (46.39) | | 0.013 | |
| Beta-blocker | 7559 | (35.40) | 3821 | (33.5) | 3738 | (37.56) | 0.085 |  | 5743 | (35.64) | 2809 | (34.86) | 2934 | (36.42) | | 0.032 | |
| Ivabradine | 28 | (0.13) | 19 | (0.17) | 5 | (0.05) | 0.035 |  | 9 | (0.06) | 4 | (0.05) | 5 | (0.06) | | 0.005 | |
| MRA | 4131 | (19.34) | 2406 | (21.10) | 1725 | (17.33) | 0.096 |  | 2953 | (18.33) | 1486 | (18.44) | 1467 | (18.21) | | 0.006 | |
| Statin | 3990 | (18.68) | 1941 | (17.02) | 2049 | (20.59) | 0.092 |  | 3020 | (18.74) | 1454 | (18.05) | 1566 | (19.44) | | 0.036 | |
| Diuretics | 11122 | (52.08) | 6084 | (53.35) | 5038 | (50.63) | 0.054 |  | 8281 | (51.39) | 4152 | (51.53) | 4129 | (51.25) | | 0.006 | |
| Digoxin | 2841 | (13.30) | 1684 | (14.77) | 1157 | (11.63) | 0.093 |  | 2011 | (12.48) | 1021 | (12.67) | 990 | (12.29) | | 0.012 | |
| H-ISDN | 631 | (2.95) | 365 | (3.20) | 266 | (2.67) | 0.031 |  | 461 | (2.86) | 239 | (2.97) | 222 | (2.76) | | 0.013 | |
| Antiplatelet | 9860 | (46.17) | 5201 | (45.60) | 4659 | (46.82) | 0.024 |  | 7357 | (45.66) | 3670 | (45.55) | 3687 | (45.76) | | 0.004 | |
| Anticoagulant | 1747 | (8.18) | 977 | (8.57) | 770 | (7.74) | 0.030 |  | 1314 | (8.15) | 648 | (8.04) | 666 | (8.27) | | 0.008 | |
| Antiarrhythmic | 2695 | (12.62) | 1616 | (14.17) | 1079 | (10.84) | 0.101 |  | 1938 | (12.03) | 981 | (12.18) | 957 | (11.88) | | 0.009 | |
| OHA | 6710 | (31.42) | 3216 | (28.20) | 3494 | (35.11) | 0.149 |  | 5115 | (31.74) | 2465 | (30.59) | 2650 | (32.89) | | 0.049 | |
| Insulin | 4463 | (20.90) | 2159 | (18.93) | 2304 | (23.15) | 0.104 |  | 3386 | (21.01) | 1634 | (20.28) | 1752 | (21.75) | | 0.036 | |
| NSAID | 5329 | (24.95) | 2829 | (24.80) | 2500 | (25.12) | 0.007 |  | 3984 | (24.72) | 2001 | (24.84) | 1983 | (24.61) | | 0.005 | |
| Steroid | 6080 | (28.47) | 3849 | (33.75) | 2231 | (22.42) | 0.254 |  | 4197 | (26.05) | 2167 | (26.90) | 2030 | (25.20) | | 0.039 | |
| Colchicine | 1293 | (6.05) | 771 | (6.76) | 522 | (5.25) | 0.064 |  | 905 | (5.62) | 458 | (5.68) | 447 | (5.55) | | 0.006 | |

ASMD= absolute standardized mean difference; MI= myocardial infarction; PCI= percutaneous coronary intervention; CABG= coronary artery bypass graft; CRT= cardiac resynchronization therapy; ICD= implantable cardioverter-defibrillator; ICH= intracerebral hemorrhage; CKD= chronic kidney disease; ESRD= end-stage renal disease; COPD= chronic obstructive pulmonary disease; ACEI= angiotensin-converting enzyme inhibitors; ARB= angiotensin receptor blockers; ARNI= angiotensin receptor-neprilysin inhibitor; MRA= mineralocorticoid receptor antagonists; H-ISDN= hydralazine–isosorbide dinitrate; OHA= oral hypoglycemic agents; NSAID= non-steroidal anti-inflammatory drugs

**Supplementary table S6. Time to event of pneumonia vs. UTI**

|  | Time to event, days (mean±SD) | | |
| --- | --- | --- | --- |
|  | All | Pneumonia | Urinary Tract Infection |
| Mortality | 623.06 ± 659.55 | 546.94 ± 609.92 | 703.70 ± 699.49 |
| HHF | 473.64 ± 555.41 | 450.48 ± 541.20 | 497.93 ± 569.01 |
| MI | 685.06 ± 654.28 | 700.83 ± 648.99 | 672.35 ± 659.77 |
| Stroke | 772.21 ± 735.16 | 768.94 ± 757.69 | 774.88± 716.72 |
| Primary composite endpoint | 564.20 ± 624.57 | 520.95 ± 599.07 | 608.37 ± 646.63 |

|  | **Supplementary table S7. Outcome assessment adjusted by length of infection admission, infection vs. control** | | | | | | | | |
| --- | --- | --- | --- | --- | --- | --- | --- | --- | --- |
|  | | Before adjustment for length of infection | | | | After adjustment for length of infection | | | |
|  | | Adjusted HR  (95%CI) | *p* value | Adjusted SHR (95%CI) | *p* value | Adjusted HR  (95%CI) | *p* value | Adjusted SHR (95%CI) | *p* value |
| Primary composite endpoint | | 1.760  (1.714-1.807) | <0.001 |  |  | 1.574  (1.525-1.625) | <0.001 |  |  |
| Mortality | | 1.587  (1.540-1.636) | <0.001 |  |  | 1.345  (1.298-1.394) | <0.001 |  |  |
| HHF | | 1.993  (1.922-2.066) | <0.001 | 1.960  (1.891- 2.033) | <0.001 | 1.985  (1.900-2.073) | <0.001 | 2.127  (2.035- 2.223) | <0.001 |
| AMI | | 1.332  (1.224-1.450) | <0.001 | 1.228  (1.128-1.337) | <0.001 | 1.431  (1.287-1.591) | <0.001 | 1.444  (1.301-1.603) | <0.001 |
| Ischemic stroke | | 1.769  (1.664-1.882) | <0.001 | 1.569  (1.475-1.668) | <0.001 | 1.808  (1.678-1.948) | <0.001 | 1.777  (1.648-1.915) | <0.001 |

HR= hazard ratio, SHR=Subdistribution hazard ratio (By competing risk model).

**Supplementary Figure S1. Sensitivity analysis of unmeasured confounders**


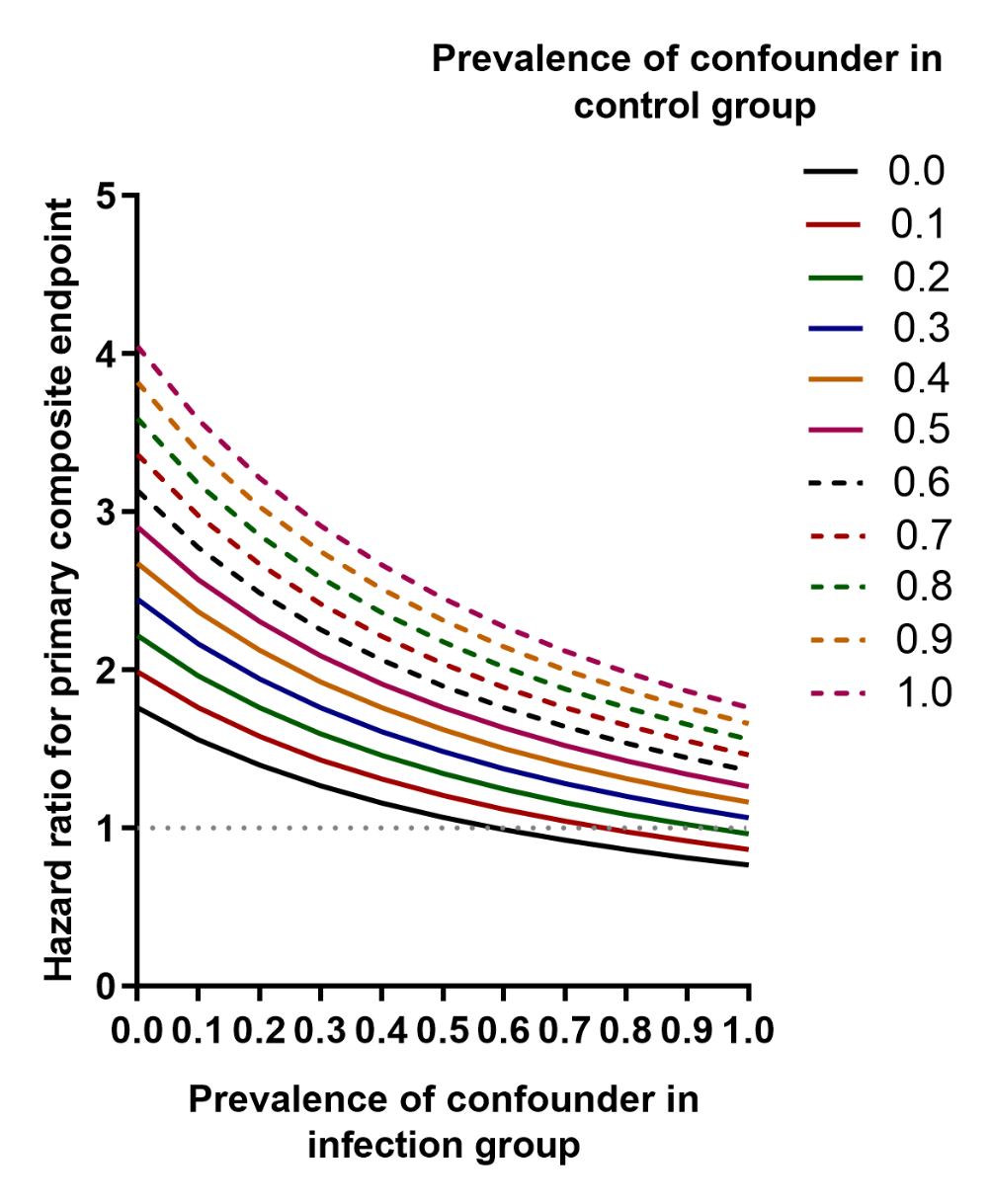


Sensitivity analysis with add-on of an unmeasured confounder for primary composite endpoint. This figure displays the trend estimates of infection group for the hazard on covariate-adjusted Cox proportional hazards model.
